# The Influence of County Contexts on Preterm Births Among Latina Mothers in the US

**DOI:** 10.1007/s40615-025-02400-1

**Published:** 2025-03-24

**Authors:** Ezinne Nwankwo, May Sudhinaraset

**Affiliations:** 1 USC Equity Research Institute, Los Angeles, CA USA; 2 Department of Community Health Sciences, UCLA Fielding School of Public Health, Los Angeles, CA USA

**Keywords:** Preterm birth, Latino density, Immigrant concentration, Residential contexts, Ethnic enclaves, Counties

## Abstract

Preterm birth (PTB), defined as delivery before 37 weeks of gestation, is a significant public health issue, with implications for newborns, families, and society. The residential contexts in which mothers live may, in part, shape their risk of delivering preterm. Although ethnic enclaves—residential settings with a large concentration of people from the same ethnic group—exist within counties, little is known about how different county contexts shape PTB. This national study investigates the association between living in distinct county types and preterm births among Latina mothers (*N* = 1,084,867). Data are from 2 years (2017–2018) of US birth records merged with demographic and policy datasets. A county-level classification scheme that integrates social (i.e., Latino ethnic density, immigrant concentration), economic (i.e., socioeconomic indicators), and geography-related (i.e., suburban and urban) county characteristics was used to represent different areas. We fit multiple logistic regression models, adjusting for individual, community, and structural-level covariates, and found significant differences in PTB across county types (*p* < 0.001). In fully adjusted models, Latina mothers in almost all county types reported 5–25% higher odds of PTB than their counterparts in suburban counties with medium Latino concentration, high immigrant density, and low economic disadvantage. These areas were distinct from the high-disadvantage suburban counties with a large share of Latinos and immigrants, where Latina mothers experienced the highest likelihood of PTB (OR = 1.25, 95% CI 1.20, 1.30). County-level social, economic, and geography-related determinants interact, shaping Latina mothers’ PTB risk. To ensure healthy environments for all, initiatives that bolster local communities are needed.

## Introduction

Preterm birth (PTB)—defined as delivery before 37 weeks of gestation—is a significant public health issue with short and long-term implications for newborns, families, and society [1–4]. In the US, about 1 in 10 babies are born preterm, with Black, Native American, and Pacific Islander women experiencing higher preterm birth rates than other groups [5, 6]. These disparities extend to Latina mothers, whose preterm birth rates vary by Latino origin, nativity, and documentation status [5, 7–9]. Although the causes of preterm births are multifaceted, resulting from structural, social, physiological, and behavioral influences [4], the likelihood of experiencing a preterm birth may depend, in part, on the residential contexts in which Latina mothers live.

Research on residential contexts among Latina mothers has often focused on ethnic enclaves, defined as geographic areas with a large concentration of people from the same ethnic group [10–12]. Although these studies suggest that living in an ethnic enclave provides protective health benefits, including reducing exposure to interpersonal racism, increasing access to culturally specific resources and social support, and reinforcing health-promoting cultural norms, research on birth outcomes has often yielded inconsistent results [9, 12–14]. Whereas some studies find that Latina mothers who live in ethnic enclaves experience an increased likelihood of preterm birth [15], gestational diabetes [16], and infant mortality [17], others report that living in such settings lessens maternal smoking [18], low weight birth [19], and infant death [18, 20]. While these effects may stem from proximal processes within the enclave, they may also result from the broader county context in which enclaves are located [9, 21, 22].

Across the US, ethnic enclaves exist in counties that differ not only in demographics, population density, diversity, or land area, but also in their ability to provide health-related programs and services that directly impact residents’ health [23–25]. While some counties are well-resourced—in terms of their economic strength, public services, community resources, and health infrastructure—others face substantial constraints in meeting residents’ needs [24, 26]. Well-resourced counties may expand access to healthcare and other services, while less-resourced counties may lack the capacity to do so [24]. County contexts may therefore disparately influence PTB risk among Latina mothers.

In a recently outlined classification, counties are organized into distinct types using the social environment, the economic context, and geography [21, 22]. The social environment, conceptualized as the relationships and interactions between people, includes social support, social networks, and social capital [27]. The classification uses the share of Latinos (i.e., ethnic density) and the share of immigrants (i.e., immigrant concentration) living in a county to highlight the role of bonding and bridging social capital. While bonding social capital may be derived from relationships with people from similar backgrounds or close relatives and friends, bridging social capital links people who may not otherwise be connected [28, 29].

In addition, social capital varies across US counties and shapes the level of trust, cohesion, and reciprocity in communities [30]. Counties with a large share of coethnic residents may have more bonding capital or ties that are associated with social support [28, 31]. In such settings, support networks may buffer stressful experiences, reducing the likelihood of preterm births [32, 33]. On the other hand, places with fewer coethnic or immigrant residents may have limited opportunities to form supportive relationships with people who share their background, which may result in stress and isolation—increasing preterm birth risk [33]. In areas with a moderate share of coethnics, residents may benefit from proximity to ethnic and immigrant networks while also accessing resources and opportunities outside these relationships. Thus, access to both forms of capital may be beneficial during pregnancy and may reduce the likelihood of preterm birth [32]. Even so, economic conditions may determine the extent to which residents leverage their shared ties [34, 35]. Those facing economic challenges may be unable to assist others in their network, which can impact perceived support and cohesion, ultimately increasing stress and preterm birth risk [34, 35].

The economic context, especially the availability, quality, and investments in employment, education, public services, and related infrastructure, shapes the circumstances in which residents live and the resulting health impacts [36, 37]. Preterm births may result from systemic racial inequities, which limit access to short and long-term economic opportunities (e.g., living wage, wealth accumulation), but may also arise from the persistent challenges that constrain access to basic needs and material resources (e.g., housing burden, food insecurity) [37, 38]. Previous studies have linked county-level economic conditions to birth outcomes, finding that pregnant women in economically strong counties are less likely to deliver preterm or deliver low birth-weight infants [39, 40]. Compared to their counterparts in affluent areas, residents in systematically neglected counties may face underfunded services, economic instability, and barriers to quality prenatal and maternal care [41].

The economic context is also shaped by county-level spatial characteristics such as urban or suburban geography. Specifically, the distribution of land, people, and resources within counties impacts residents’ access to opportunities and services [26, 42, 43]. Compared to Latina residents in urban areas who may have opportunities to build a community because of their proximity to support networks, public transportation, social services, and other resources, those in dispersed suburbs may be less able to cultivate strong ties because of their more diffuse population. This social and service dispersion may result in isolation and a lack of support—risk factors for PTB [4, 44, 45].

Suburbs are also increasingly socially and economically diverse [26, 42, 43]. Unlike affluent suburbs, where residents may have access to robust services and strong social safety nets, Latina residents in economically disadvantaged suburban areas may face poverty, food insecurity, and transportation barriers that limit their interaction with prenatal care and other health and social services [26, 42, 43]. Although a large share of Latinos live in enclaves located in counties across the US [46], there is limited understanding of how disparate county contexts shape PTB risk among Latina mothers.

In this study, we used 2 years (2017–2018) of US birth records merged with census, health, and policy-related measures to investigate the association between living in distinct county types—demarcated by county-level social, economic, and geographic characteristics—and preterm births. We hypothesized that PTB risk would not only vary across county types but that relative to residents in suburban counties with a moderate share of Latinos and a large immigrant population, Latina mothers in all other county types would experience a higher likelihood of preterm birth.

## Methods

### Data Sources

Data from multiple sources were merged to create the analytic dataset. Two years (2017–2018) of restricted-use birth certificate data were obtained from the Centers for Disease Control and Preventions’ (CDC) National Center for Health Statistics (NCHS). Population data were retrieved from the US Census Bureau’s 5-year (2014–2018) American Community Survey (ACS) and the 2010 decennial census. Additional indicators were sourced from the Immigrant Legal Resource Center (ILRC), the County Health Rankings, the Massachusetts Institute of Technology’s Election Data and Science Lab, the Census of Governments, the National Conference of State Legislatures, the Kaiser Family Foundation (KFF), and the Migration Policy Institute. Data were merged using Federal Information Processing Series codes. The birth certificate data were deidentified and exempt from human subjects’ research review; all other datasets are publicly available. See Appendix Table 5 for a list of variables and their data sources.

### Sample

We applied several restrictions to obtain the final analytic sample. There were 3,864,754 births in 2017 and 3,801,534 births in 2018. We excluded births to foreign nationals (*n* = 19,076), non-Hispanic mothers (*n* = 5,795,889), and those with missing or unknown information about Latino origin (*n* = 361,062). Plural births were also excluded (*n* = 36,578) along with births to women (*n* = 1,332) outside reproductive age (15–49), births with an unknown gestation period (*n* = 681), and records where gestation age was not viable (less than 20 weeks, *n* = 384). The final sample included 1,084,867 births to Latina mothers residing in 115 counties in the US.

### Variables

The outcome variable was preterm birth, coded as full-term or preterm. The primary predictor variable was a nine-category county classification measure. The variable was constructed with data from the 2014–2018 ACS and represents 232 US counties where Latinos constitute more than 13.75% of the population. The classification is based on a stepwise process that accounts for county-level social, economic, and geography characteristics. The starting branch, the social environment, includes ethnic density (i.e., percent Latino)—divided into medium (13.75–27.49%) and high (≥ 27.49%) density—and immigrant concentration (i.e., percent foreign-born). The second branch, the geographic dimension, originated from the NCHS classification of US counties and was categorized as suburban or urban. The third branch, the economic context, was derived using a factor score of five county-level socioeconomic characteristics, including the share of residents on public assistance, children living in poverty, people with a bachelor’s degree or higher, unemployed residents, and median household income. Additional details about the classification process are described in full elsewhere [22].

Classified county types included low- and high-economic-disadvantage suburban and urban counties with a large share of Latinos and immigrants, low- and high-economic-disadvantage suburban and urban counties with a large concentration of Latinos but a small immigrant population (no low-economic-disadvantage urban counties of this type were identified), and low- and high-economic-disadvantage suburban counties with a moderate Latino population and a large share of immigrants. Table 1 presents a description of the county types used in this study.

**Table 1:** Classified county types, *N* = 232

County type	Social environment		Geography	Economic disadvantage	*n* (%)
	Latino ethnic density	Immigrant concentration			
Type 1	Large	Large	Suburban	High	29 (13.0)
Type 2	Large	Large	Suburban	Low	20 (9.0)
Type 3	Large	Large	Urban	High	11 (5.0)
Type 4	Large	Large	Urban	Low	25 (11.0)
Type 5	Large	Small	Suburban	High	41 (18.0)
Type 6	Large	Small	Suburban	Low	56 (24.0)
Type 7	Large	Small	Urban	High	2 (1.0)
Type 9	Medium	Large	Suburban	Low	5 (2.0)
Type 10	Medium	Large	Suburban	High	43 (19.0)

*Note*: Type 8 counties were not classified because no urban areas with low economic disadvantage, a large Latino population, and a small immigrant concentration were identified

### Individual-Level Factors

Individual-level covariates included age (continuous), marital status (married, unmarried, or missing), total number of live births (continuous), educational status (8th grade or less, some high school, high school grad/GED, some college/associates, college/higher or missing), and type of insurance coverage (Medicaid, private insurance, self-pay, other, or missing). Preterm birth risk factors were based on self-reports of gestational diabetes, hypertension, eclampsia, or previous PTB and were included as an aggregate measure, coded as no risk factors or one or more PTB risk factors. Prenatal care represented initiating early prenatal care or starting prenatal care after the first trimester or not at all. Smoking during pregnancy was either no or yes. Latino origin was categorized as Mexican, Puerto Rican, Cuban, or Central/South American. Nativity indicates if mothers were US or foreign-born. Based on NCHS procedures, mothers born in Puerto Rico were considered foreign-born.

### Community-Level Factors

The number of membership-based social associations (per 10,000 population) was included as a proxy of informal and formal networks and was a county-level measure.

### Structural-Level Factors

To account for contextual differences between counties, we used several variables, conceptualized as structural influences. Residential segregation, between Latino and white residents, was based on the dissimilarity and isolation indexes, which assess separation and concentration, respectively. We included a measure of income inequality, as the ratio of household income at the 80th percentile to income at the 20th percentile. Healthcare access was based on the ratio of the county population to primary care physicians and other primary care providers (e.g., nurse practitioners, physician assistants) and county-level public health expenditures.

The county-level policy context captured inclusive and exclusive immigrant policies and the share of the county electorate that voted for the Republican presidential candidate in 2016. A binary yes or no indicator was used to evaluate if the states in which counties were located covered lawful immigrant children and/or pregnant women without a 5-year wait for the Medicaid/Children’s Health Insurance Program (CHIP). To determine how involved counties were with Immigration and Customs Enforcement (ICE), we used the ILRC’s measure of local entanglement with ICE, which is based on seven questions that indicate if the county (1) has a 287 (g) agreement with ICE, (2) has a contract with ICE to detain immigrants in county detention facilities, (3) limits or refuses to hold individuals after their release date, (4) has a policy against notifying ICE of release dates and times or other information about inmate status, (5) allows ICE in the jail or requires consent from detainees before ICE agents are allowed to interrogate them while in custody, (6) prohibits asking people about their immigration status, or (7) generally prohibits providing assistance and resources to ICE for the purposes of enforcing civil immigration laws or prohibits participating in joint task forces. We recoded this score so that a 7 indicated the highest level of ICE corporation and 0 represented no involvement with ICE (mean = 2.8, SD = 2.5, range = 0–7).

Four covariates were included to account for population-level variation. These included the share of rural residents and population density for each county, the proportion of undocumented foreign-born population, and the degree of population change since 2000 for each state.

### Analyses

We investigated the distribution of study variables and then examined correlations between them; skewed continuous variables were log-transformed. In bivariate analyses, we examined differences between each variable and PTB across county types using *t*-tests and ANOVA and chi-square tests. Odds ratios (OR) were used to investigate bivariate associations. The final multivariable logistic regression model adjusts for individual, community, and structural-level covariates. After fitting this model, we conducted post hoc analyses to examine which dimension of the county-type variable was most influential (see Appendix Table 6 for results).

In sensitivity analyses, we fit a multilevel model, with county-level random effects, but observed little variation between counties due to the aggregated county-level measure. The intraclass correlation coefficient, *ρ* = 0.01, suggested that less than 1% of the variation between counties was explained by the random component. We also fit adjusted logistic regression models to evaluate how our results changed with low birth weight (birth weight of less than 2500 g) as the outcome and observed a similar overall pattern as with preterm birth. All analyses were performed using Stata v17.0, and results were deemed statistically significant at *p* < 0.05.

## Results

### Descriptive Analyses

Table 2 presents the characteristics of the study sample (*N* = 1,084,867), where about 8.3% of births were preterm (*n* = 89,867). There were statistically significant differences in the preterm birth rate by almost all study variables. With respect to county types, high-disadvantage urban counties with a large Latino presence but fewer immigrants had the highest rate of preterm births (9.7%), whereas low-disadvantage suburban counties with a large Latino and foreign-born population had the lowest rates (7.3%). Over 50% of mothers in our sample lived in urban areas with a large share of Latinos and immigrants; the majority (29.1%) lived in type 4, low-disadvantage counties.

**Table 2:** Characteristics of the study sample, by preterm birth, *N* = 1,084,867

Variables	Total^a^	Full term^b^	Preterm^b^	*p*-value
	*n* (%)	*n* (%)	*n* (%)	
Sample	1,084,867	995,000 (91.5)	89,867 (8.3)	
County type				0.00
Type 1	169,558 (15.6)	155,104 (91.5)	14,454 (8.5)	
Type 2	60,738 (5.6)	56,279 (92.7)	4,459 (7.3)	
Type 3	287,934 (26.5)	262,472 (91.2)	25,462 (8.8)	
Type 4	316,047 (29.1)	290,546 (91.9)	25,501 (8.1)	
Type 5	53,067 (4.9)	48,461 (91.3)	4,606 (8.7)	
Type 6	41,485 (3.8)	38,203 (92.1)	3,282 (7.9)	
Type 7	10,325 (1.0)	9,320 (90.3)	1,005 (9.7)	
Type 9	14,325 (1.3)	13,231 (92.4)	1,094 (7.6)	
Type 10	131,388 (12.1)	121,384 (92.4)	10,004 (7.6)	
Individual level factors				
*Nativity*				0.00
U.S. Born (ref)	551,475 (50.8)	504,622 (91.5)	46,853 (8.5)	
Foreign born	533,392 (49.2)	490,378 (91.9)	43,014 (8.1)	
*Latino origin*				0.00
Mexican	762,291 (70.3)	699,231 (91.7)	63,060 (8.3)	
Puerto Rican	87,419 (8.1)	79,003 (90.4)	8,416 (9.6)	
Cuban	36,460 (3.4)	33,733 (92.5)	2,727 (7.5)	
Central/South American	198,697 (18.3)	183,033 (92.1)	15,664 (7.9)	
* Maternal age*^c^	28.2 (6.1)	28.1 (6.1)	29.0 (6.5)	0.00
*Marital status*				0.00
Married	353,046 (32.5)	323,691 (91.7)	29,355 (8.3)	
Unmarried	387,855 (35.8)	353,327 (91.1)	34,528 (8.9)	
Missing	343,966 (31.7)	317,982 (92.4)	25,984 (7.6)	
*Education*				0.00
8th grade or less (ref)	93,187 (8.6)	85,083 (91.3)	8,104 (8.7)	
Some high school	197,678 (18.2)	179,751 (90.9)	17,927 (9.1)	
High school grad/GED	352,820 (32.5)	323,434 (91.7)	29,386 (8.3)	
Some college/Associates	280,297 (25.8)	257,320 (91.8)	22,977 (8.2)	
College +	147,275 (13.6)	136,974 (93.0)	10,301 (7.0)	
Missing	13,610 (1.3)	12,438 (91.4)	1,172 (8.6)	
*Insurance*				0.00
Medicaid (ref)	651,929 (60.1)	597,399 (91.6)	54,530 (8.4)	
Private insurance	317,824 (29.3)	292,925 (92.2)	24,899 (7.8)	
Self-pay	65,789 (6.1)	60,030 (91.2)	5,759 (8.8)	
Other	45,761 (4.2)	41,463 (90.6)	4,298 (9.4)	
Missing	3,564 (0.3)	3,183 (89.3)	381 (10.7)	
* No. of live births*^c^	2.3 (1.3)	2.3 (1.3)	2.4 (1.5)	0.00
*PTB risk factors*				0.00
No	957,802 (88.3)	888,546 (92.8)	69,256 (7.2)	
Yes	127,065 (11.7)	106,454 (83.8)	20,611 (16.2)	
*Smoked during pregnancy*				0.00
No	1,073,359 (98.9)	985,126 (91.8)	88,233 (8.2)	
Yes	11,508 (1.1)	9,874 (85.8)	1,634 (14.2)	
*Prenatal care*				0.00
Early initiation	780,820 (72.0)	716,842 (91.8)	63,978 (8.2)	
2nd tri/Late/no initiation	304,047 (28.0)	278,158 (91.5)	25,889 (8.5)	
Community-level factors				
Social associations^c^	6.5 (2.0)	6.5 (2.0)	6.5 (2.0)	0.02
Structural factors				
*Five-year waiting period*				0.00
No	546,810 (50.4)	499,052 (91.3)	47,758 (8.7)	
Yes	538,057 (49.6)	495,948 (92.2)	42,109 (7.8)	
Isolation index^c^	51.3 (16.4)	51.3 (16.3)	51.8 (16.7)	0.00
Dissimilarity index^c^	47.3 (9.7)	47.2 (9.7)	47.4 (9.6)	0.00
Income inequality^c^	4.9 (0.7)	4.9 (0.7)	5.0 (0.7)	0.00
Primary care physicians^c^	1462.9 (508.5)	1463.1 (509.9)	1460.1 (493.6)	0.09
Other primary care^c^	1447.3 (490.9)	1449.7 (492.1)	1421.5 (476.6)	0.00
Public health expenditure (million $)^c^	760 (1400)	760 (1400)	740 (1400)	0.00
Immigration enforcement score (0–7)^c^	2.8 (2.5)	2.8 (2.5)	2.9 (2.5)	0.00
%County population voting Republican (2016)^c^	37.2 (13.2)	37.2 (13.2)	37.1 (13.2)	0.07
%Population change since 2000^c^	21.6 (11.6)	21.6 (11.6)	22.0 (11.8)	0.00
%Rural population in county^c^	4.5 (6.4)	4.5 (6.4)	4.4 (6.2)	0.00
State undocumented immigrant population^c^	27.4 (6.6)	27.4 (6.6)	27.8 (6.7)	0.00
Population density^c^	2661.1 (3490.4)	2667.4 (3492.8)	2591.4 (3463.7)	0.00

^a^Column percentages or the total sample distribution across categories of individual variables

^b^Row percentages or the total sample distribution across full-term and preterm birth

^c^Mean values and standard deviation

There was a near-equal distribution of US (50.8%) and foreign (49.2%) born mothers in this sample, with immigrant mothers experiencing lower rates of preterm births (8.1%) than their US-born counterparts (8.5%). Mexican-origin mothers (70.3%) represented the largest group in our sample. However, across Latina subgroups, Cuban-origin mothers had the lowest preterm birth rate (7.5%); preterm birth rates were highest among mothers of Puerto Rican descent (9.6%). Mothers who experienced a preterm birth tended to be older than their counterparts who did not (*M* = 29, SD = 6.5 vs. *M* = 28.1, SD = 6.1), and those who were either unmarried (8.9%) or only completed some high school (9.1%) experienced higher preterm birth rates. Most births were covered by Medicaid (60.1%), with mothers who reported being privately insured experiencing lower preterm birth rates (7.8%) than those who reported other forms of payment for their delivery.

There were significant differences in preterm birth rates across several health and behavioral risk factors. Mothers that delivered preterm were significantly more likely to have had 2.4 (SD = 1.5) previous live births than their counterparts (*M* = 2.3, SD = 1.3) who delivered full term. Of the 11.7% of the sample that reported one or more preterm birth risk factors (i.e., having gestational diabetes, hypertension, eclampsia, or previous premature birth), 16.2% of them delivered too early. While few mothers disclosed smoking during pregnancy (1.1%), about 14.2% of those who smoked experienced a preterm delivery. Of the 28.0% of mothers who sought prenatal care later than recommended, 8.5% delivered preterm.

While most people in this sample lived in counties with six or more social associations per 10,000 people (range = 0–21.1), there were no quantitative differences in the number of social associations (*M* = 6.5, SD = 2.0) when comparing birth outcomes. Of the 49.6% of mothers who lived in states that covered lawful pregnant immigrants without a 5-year eligibility period, 7.8% of them experienced a preterm delivery. Latina mothers who delivered preterm were slightly less likely to be isolated from other Latinos (*M* = 51.3, SD = 16.3 vs. *M* = 51.8, SD = 16.7), but marginally more likely to live in places with greater distributional dissimilarity between Latinos and white residents (*M* = 47.2, SD = 9.7 vs. *M* = 47.4, SD = 9.6).

Latina mothers who lived in counties with greater income inequality were significantly more likely to deliver preterm (*M* = 4.9, SD = 0.7 vs. *M* = 5.0, SD = 0.7), and those that experienced a preterm birth were more likely to live in counties with smaller ratios of residents to primary care providers (*M* = 1463.1, SD = 509.9 vs. *M* = 1460.1, SD = 493.6) and other primary care practitioners (*M* = 1449.7, SD = 492.1 vs. *M* = 1421.5, SD = 476.6). These mothers were also more likely to live in areas that received about 20 million dollars less in public health expenditures. Although most people lived in counties with about 2.8 (SD = 2.5) immigration enforcement policies, mothers that delivered preterm were significantly more likely to live in areas with slightly more of these measures (*M* = 2.8, SD = 2.5 vs. *M* = 2.9, SD = 2.5).

Latina mothers who delivered preterm were significantly more likely to live in states that experienced a 22% (SD = 11.8) average population increase since 2000. These mothers were also more likely to live in counties with a smaller rural population (*M* = 4.5, SD = 6.4 vs. *M* = 4.4, SD = 6.2). The counties in this study were generally in states where over one in four immigrants were undocumented (*M* = 27.4, SD = 6.6), and those who experienced premature births were more likely to live in such states (*M* = 27.4, SD = 6.6). Population density was significantly lower in places where mothers delivered preterm (*M* = 2667.4, SD = 3492.8 vs. *M* = 2591.4, SD = 3463.7).

### Bivariate Analyses

There were significant statistical differences between county types on all study measures (Table 3). While individual-level characteristics in each county type generally reflected that of the overall sample, there were some notable differences. For instance, although Mexican-origin mothers were more likely to represent over 60% of Latino residents in almost all other county types, they represented less than 50% of people in suburban counties with a moderate share of Latinos and a large immigrant population (type 10 (40.9%) and type 9 (47.8%)). The low-disadvantage suburbs (type 10) were primarily home to Central/South American origin mothers (42.8%); Puerto Rican (23.0%) and Central/South American origin mothers (26.4%) were well-represented in the high-disadvantage suburbs (type 9).

**Table 3:** Distribution of study variables by county type (%), *N* = 1,084,867

Variables	Type 1	Type 2	Type 3	Type 4	Type 5	Type 6	Type 7	Type 9	Type 10	*p*-value
Preterm birth										
37 + weeks	91.5	92.7	91.2	91.9	91.3	92.1	90.3	92.4	92.4	
Under 37 weeks	8.5	7.3	8.8	8.1	8.7	7.9	9.7	7.6	7.6	
Individual level factors										
*Nativity status*										***
U.S. Born	56.8	43.9	49.0	53.9	62.1	54.4	48.2	44.8	38.1	
Foreign born	43.2	56.1	51.0	46.1	37.9	45.6	51.8	55.2	61.9	
*Ethnicity*										***
Mexican	92.4	70.1	61.3	77.7	76.4	77.6	75.3	47.8	40.9	
Puerto Rican	2.8	4.2	10.4	5.0	16.5	8.8	14.3	23.0	13.1	
Cuban	0.4	7.5	7.8	0.9	1.3	0.9	0.7	2.7	3.3	
Central/South American	4.4	18.2	20.4	16.4	5.8	12.7	9.8	26.4	42.8	
* Maternal age*^a^	27.5 (6.0)	28.5 (6.1)	28.3 (6.1)	28.6 (6.1)	26.9 (5.9)	27.5 (6.0)	27.5 (6.2)	27.4 (6.0)	28.8 (6.2)	***
*Marital status*										***
Married	25.3	29.4	41.4	20.1	44.7	45.8	46.2	42	42.5	
Unmarried	27.3	23.7	47.6	21.8	52.8	43.8	53.8	58	46.6	
Missing	47.5	46.9	11.0	58	2.5	10.3	0.0	0.0	10.9	
*Education*										***
8th grade or less	7.7	12.6	6.4	8.1	7.3	8.8	11.0	11.8	13.9	
Some high school	18.6	16.4	19.2	17.2	20.3	19.5	21.9	19.2	17.1	
High school grad/GED	33.4	29.0	34.4	31.6	34.4	34.4	34.3	34.8	29.2	
Some college/Associates	28.0	26.0	24.1	27.0	28.4	26.2	21.8	24.7	23.3	
College +	10.7	14.8	14.7	14.8	9.3	10.2	10.7	8.2	14.9	
Missing	1.5	1.2	1.2	1.3	0.3	0.9	0.3	1.3	1.5	
*Insurance*										***
Medicaid	63.5	59.4	58.8	59.6	57.6	63.8	76.7	65.9	57.9	
Private insurance	24.1	31.4	28.8	32.2	25.6	27.7	20.8	23.3	32.5	
Self-pay	7.6	6.4	6.5	4.6	8.8	3.5	0.9	5.0	6.8	
Other	4.5	2.7	5.5	3.3	7.8	4.3	1.2	5.3	2.5	
Missing	0.3	0.1	0.4	0.3	0.3	0.6	0.3	0.5	0.4	
* Live birth order*^a^	2.4 (1.4)	2.2 (1.3)	2.2 (1.3)	2.2 (1.3)	2.4 (1.4)	2.3 (1.3)	2.5 (1.5)	2.4 (1.3)	2.2 (1.3)	***
*Preterm birth risk factors*										***
No	90.8	86.9	88.0	88.2	86.9	86.9	88.4	86	87.7	
Yes	9.2	13.1	12.0	11.8	13.1	13.1	11.6	14	12.3	
*Smoked during pregnancy*										***
No	99.1	99.2	99.2	99.3	96.6	97.7	96.7	96.8	99.0	
Yes	0.9	0.8	0.8	0.7	3.4	2.3	3.3	3.2	1.0	
*Prenatal care initiation*										***
Early initiation	71.2	72	68.3	78.3	66.9	69.4	69.3	68.9	69.3	
2nd tri/Late/no initiation	28.8	28	31.7	21.7	33.1	30.6	30.7	31.1	30.7	
Community-level factors										
Social associations^a^	5.1 (1.4)	6.5 (1.7)	5.8 (1.4)	6.5 (1.9)	9.1 (2.3)	7.9 (1.5)	9.7 (1.0)	9.4 (2.5)	7.5 (1.5)	***
Structural factors										
*Covered without waiting period*										***
No	47.6	42.9	80.1	27.7	69.4	39.0	57.0	63.4	41.0	
Yes	52.4	57.1	19.9	72.3	30.6	61.0	43.0	36.6	59.0	
Isolation index^a^	69.7 (15.7)	52.0 (12.6)	55.2 (12.3)	51.3 (11.3)	38.1 (13.9)	30.0 (7.8)	40.2 (0.8)	33.5 (9.0)	33.4 (5.2)	***
Dissimilarity index^a^	41.7 (6.3)	44.7 (11.1)	49.0 (8.1)	53.4 (7.4)	38.8 (11.2)	34.9 (9.4)	53.7 (0.4)	43.9 (9.7)	44.2 (8.1)	***
Income inequality^a^	4.9 (0.5)	4.5 (0.5)	5.1 (0.6)	5.1 (0.9)	4.7 (0.5)	4.6 (0.6)	4.8 (0.2)	4.6 (0.4)	4.7 (0.6)	***
Primary care physicians^a^	2001.2 (582.1)	1551.1 (508.6)	1470.7 (348.6)	1264.2 (259.3)	1646.8 (901.7)	1453.4 (508.0)	1259.9 (90.7)	1436.7 (539.1)	1135.6 (333.6)	***
Other primary care^a^	1634.8 (493.4)	1645.0 (620.7)	1300.2 (358.7)	1533.0 (504.8)	1166.9 (484.4)	1342.4 (470.5)	690.9 (84.5)	1140.1 (426.7)	1469.7 (444.9)	***
Public health expenditure (million $)^a^	760 (1500)	210 (240)	320 (260)	1800 (1900)	30 (20)	54 (45)	200 (220)	43 (34)	120 (98)	***
Immigration enforcement score (0–7)^a^	2.6 (2.6)	2.5 (2.7)	3.5 (2.3)	1.6 (2.2)	4.5 (1.9)	3.6 (2.5)	4.1 (1.0)	3.4 (1.9)	3.3 (2.2)	***
%County pop. voting Republican-2016^a^	40.3 (11.7)	36.3 (12.0)	34.9 (9.2)	31.0 (12.6)	54.8 (12.0)	52.7 (12.7)	41.8 (11.4)	49.2 (4.9)	39.9 (12.9)	***
%Population change since 2000^a^	23.9 (9.2)	20.2 (8.5)	24.9 (13.3)	19.8 (10.1)	23.2 (11.5)	24.7 (10.4)	11.0 (2.9)	15.5 (12.0)	16.1 (12.3)	***
%Rural pop. in county^a^	8.8 (5.8)	5.9 (6.1)	1.6 (1.7)	1.4 (1.5)	16.5 (10.9)	14.8 (10.2)	3.7 (3.0)	8.0 (4.6)	3.9 (4.7)	***
%State Hispanic pop^a^	36.4 (7.0)	31.3 (8.8)	30.7 (9.3)	34.4 (8.1)	28.5 (14.0)	26.5 (11.8)	8.8 (1.8)	15.6 (10.8)	22.9 (10.1)	***
%State foreign-born pop^a^	21.2 (5.8)	21.7 (5.7)	18.8 (4.0)	22.4 (5.9)	14.2 (5.5)	15.4 (6.2)	5.6 (0.5)	11.6 (6.3)	18.9 (4.6)	***
State undocumented immigrant pop^a^	29.3 (5.7)	23.9 (5.3)	27.7 (7.9)	26.8 (4.8)	30.4 (7.4)	31.4 (6.5)	33.4 (6.7)	26.4 (7.3)	24.2 (5.8)	***
Population density^a^	5348.0 (6165.3)	1683.1 (880.5)	2118.3 (2313.5)	3194.5 (2840.9)	1470.5 (2299.1)	1249.0 (1268.4)	507.9 (231.4)	620.0 (355.4)	870.8 (1032.7)	***

Note: ^a^*M* mean, *SD* standard deviation, *Pop*. population

In addition, the number of social associations varied between counties, ranging from 5.1 (SD = 1.4) in high-disadvantage suburban counties with large shares of Latinos and immigrants to 9.7 (SD = 1.0) in urban counties with large Latino populations but fewer foreign-born residents. Latina mothers who lived in low-disadvantage urban counties with fewer Latinos and immigrants were more likely to live in states that covered lawful immigrants without a 5-year waiting period (72.3%). Income inequality was most pronounced in urban counties, especially high (type 3, *M* = 5.1, SD = 0.6) and low (type 4, *M* = 5.1, SD = 0.9) disadvantage areas with large shares of Latinos and immigrants.

Latina mothers in high-disadvantage suburban counties with a large representation of Latinos and immigrants were the most likely to be exposed to other Latino people (*M* = 69.7, SD = 15.7). However, those in urban counties characterized by high disadvantage and large shares of Latinos but fewer immigrants faced the highest levels of residential segregation. In these counties, over 50% of Latinos would need to move to ensure an even Latino-white population distribution (*M* = 53.7, SD = 0.4). Although the average share of the undocumented population was over 20% across county types, these counties were in states that had more undocumented individuals (*M* = 31.4, SD = 6.5).

While low-disadvantage urban counties with large shares of Latinos and immigrants had the fewest immigration enforcement policies (*M* = 1.6, SD = 2.2), high-disadvantage suburban counties with large shares of Latinos but fewer immigrants (type 5) had the most of these policies (*M* = 4.5, SD = 1.9). Residents in type 5 counties were also more likely to vote for the Republican candidate in the 2016 election (*M* = 54.8, SD = 12.0). These counties also spent less on public health (*M* = $30 million, SD = $20 million) and had the largest share of rural residents (*M* = 16.5, SD = 10.9).

Primary care physicians and other primary care providers in high (type 1)- and low (type 2)-disadvantage suburban areas with large shares of Latinos and immigrants typically had to serve more people. Type 1 counties had the largest ratio of the population to primary care physicians (*M* = 2001.2, SD = 582.1), and type 2 counties had the largest ratio of the population to other primary care providers (*M* = 1645.0, SD = 620.7).

### Multivariable Analyses

Results from the multivariable logistic regression models predicting preterm birth are outlined in Table 4. After adjusting for covariates, Latina mothers who lived in almost all other county types experienced significantly higher odds of PTB than their counterparts in low-disadvantage suburban counties with medium Latino density and a large foreign-born presence.

**Table 4:** Logistic regression results predicting the association between county type and preterm births among Latina mothers in the U.S., *N* = 1,084,867

Variables	aOR	95% CI
County type		
Type 1	1.25***	1.20, 1.30
Type 2	1.08***	1.04, 1.13
Type 3	1.10***	1.07, 1.14
Type 4	1.05**	1.01, 1.08
Type 5	1.12***	1.07, 1.17
Type 6	1.05*	1.01, 1.10
Type 7	1.16***	1.07, 1.25
Type 9	0.99	0.92, 1.06
Type 10 (ref)	1.00	1.00, 1.00
Individual level factors		
*Nativity*		
U.S. Born (ref)	1.00	1.00, 1.00
Foreign born	0.85***	0.84, 0.86
*Hispanic origin*		
Mexican (ref)	1.00	1.00, 1.00
Puerto Rican	1.23***	1.19, 1.26
Cuban	0.98	0.94, 1.03
Central/South American	1.04***	1.02, 1.07
*Age*		
Maternal age (cont.)	1.02***	1.02, 1.02
*Marital status*		
Married (ref)	1.00	1.00, 1.00
Unmarried	1.10***	1.08, 1.12
Missing	1.03	0.99, 1.08
*Education*		
8th grade or less (ref)	1.00	1.00, 1.00
Some high school	1.08***	1.05, 1.12
High school grad/GED	1.01	0.98, 1.04
Some college/Associates	0.96**	0.93, 0.98
College +	0.78***	0.76, 0.81
Missing	1.08*	1.01, 1.15
*Insurance*		
Medicaid (ref)	1.00	1.00, 1.00
Private insurance	0.98*	0.96, 1.00
Self pay	1.08***	1.05, 1.11
Other	1.11***	1.07, 1.15
Missing	1.38***	1.24, 1.54
*No. of live births*		
Live birth order	1.03***	1.02, 1.03
* Risk factors*		
No (ref)	1.00	1.00, 1.00
Yes	2.39***	2.35, 2.43
*Smoking*		
No (ref)	1.00	1.00, 1.00
Yes	1.60***	1.51, 1.69
*Prenatal care*		
Early initiation (ref)	1.00	1.00, 1.00
2nd tri/Late/no initiation	1.02*	1.00, 1.03
Community-level factors		
Social associations (log)	0.99	0.94, 1.04
Structural factors		
Isolation index	0.97***	0.95, 0.99
Dissimilarity index	1.04	0.93, 1.17
Income inequality (log)	1.49***	1.35, 1.64
Primary care physicians (log)	0.93***	0.89, 0.96
Other primary care (log)	0.99	0.96, 1.02
Public health expenditure (log)	1.01**	1.00, 1.02
*Covered without waiting period*		
No (ref)	1.00	1.00, 1.00
Yes	0.99	0.96, 1.02
Immigration enforcement score	1.03***	1.02, 1.03
% of county pop. voting Republican, 2016 (log)	1.05**	1.01, 1.08
% State population change, 2000–2018 (log)	0.98*	0.96, 1.00
% Rural population (log)	0.97***	0.95, 0.98
% of undocumented pop. in state (log)	1.32***	1.26, 1.38
Population density (log)	1.01	1.00, 1.02
Constant	0.01***	0.01, 0.01
* N*	1,084,867	

^*^p < 0.05

^**^p < 0.01

^***^p < 0.001

+ *p* < 0.10

Relative to US-born mothers, immigrant mothers experienced 15% lower odds of PTB (aOR = 0.85, 95% CI 0.84, 0.86). Mothers of Puerto Rican origin (aOR = 1.23, 95% CI 1.19, 1.26) and those of Central/South American origin (aOR = 1.04, 95% CI 1.02, 1.07) experienced significantly higher odds of PTB than their Mexican origin counterparts.

For every year increase in age, Latina mothers experienced a 2% higher likelihood of PTB. Compared to those who were married, those who were not had 10% increased odds of PTB. Education had some protective effects, as completing some college/associate’s degree (aOR = 0.96, 95% CI 0.93, 0.98) or having a bachelor’s degree or higher (aOR = 0.78, 95% CI 0.76,0.81) was associated with significantly lower odds of PTB when all other factors were held constant. Compared to Latina mothers who reported Medicaid as their payment source, those who self-paid (aOR = 1.08, 95% CI 1.05,1.11), used some other insurance source (aOR = 1.11, 95% CI 1.07, 1.15), and those for whom this response was missing (aOR = 1.38, 95% CI 1.24, 1.54) reported a significantly higher likelihood of PTB.

Net of covariates, health, and behavioral risk factors increased Latina mothers’ likelihood of PTB, and each additional previous live birth was associated with a 3% increase in the odds of giving birth prematurely. Relative to Latina mothers who did not report gestational diabetes, hypertension, eclampsia, or a previous PTB delivery, those who did were 139% times more likely to deliver preterm. Smoking also significantly increased this risk by 60% and delaying prenatal care resulted in a 2% increase in the odds of PTB.

Latino residents increased exposure to other Latinos in the county (the isolation index) resulted in a significantly lower likelihood of delivering preterm (aOR = 0.97, 95% CI 0.95, 0.99). However, living in counties with increasing levels of income inequality was associated with 49% higher odds of PTB. While Latina mothers’ greater access to primary care physicians resulted in a significantly lower likelihood of PTB (aOR = 0.93, 95% CI, 0.89, 0.96), increased public health expenditure at the county level did not decrease this likelihood (aOR = 1.01, 95% CI 1.00, 1.02).

Living in counties with increased immigration enforcement policies (aOR = 1.03, 95% CI 1.02, 1.03) or in places where a greater share of residents who voted for the Republican candidate in the 2016 presidential election (aOR = 1.05, 95% CI 1.01, 1.08) was associated with a significantly higher likelihood of preterm delivery. There were also county-level population-related effects, as those who lived in states that experienced greater population change between 2000 and 2018 were significantly more likely to report lower odds of PTB (aOR = 0.98, 95% CI 0.96, 1.00). However, living in states where an increasing share of the population was undocumented resulted in 32% higher odds of PTB (aOR = 1.32, 95% CI 1.26, 1.38). Residing in counties with a greater share of rural residents resulted in Latina mothers experiencing a significantly lower likelihood of this outcome (aOR = 0.97, 95% CI 0.95,0.98).

## Discussion

The goal of this study was to investigate the association between living in different types of counties and preterm births among Latina mothers in the US. We used a nine-category county classification, demarcating social, economic, and geography-related characteristics, and found statistically significant differences in the likelihood of preterm births based on where mothers lived. Ultimately, Latina mothers who lived in almost all other county types experienced significantly higher odds of preterm births than their counterparts in affluent suburban counties with a moderate share of Latinos and a large immigrant population. Mothers in these more ethnically heterogenous counties may have benefited from bonding and bridging social capital, allowing them access to the health and social support resources that buffer stress, before and during pregnancy [33]. Although health research on suburban communities is still nascent, our results are in line with at least one other study that finds access to both forms of social capital may contribute to improved birth outcomes [32].

Another noteworthy finding is that Latina mothers in suburban counties that were characterized by a large concentration of Latinos and immigrants and high economic disadvantage were the most likely group to experience preterm birth. Multiple factors likely contributed to this result. It is possible that overreliance on coethnic network members for support strains existing bonds and limits the mutuality often associated with coethnic density [47]. Prior studies have found that increased social ties may not engender greater social cohesion in communities experiencing systemic disadvantages [34]. These counties also had the largest ratio of people to primary care physicians, which may have contributed to the higher likelihood of preterm birth that we observed. Latina mothers in these areas may have been unable to access pregnancy-related services because of the challenges some suburbs face, including poverty, resource deprivation, limited transportation infrastructure, and constrained access to health and organizational supports [42, 43, 48–50].

In addition, since residential segregation, not just residential preference, concentrates Latinos and immigrants into poorer communities, the much higher likelihood of preterm birth in counties facing economic challenges may reflect the joint influence of the social and economic contexts. Compared to their counterparts in more prosperous counties, Latina mothers in suburban and urban counties with a large concentration of Latinos and immigrants (type 1 and type 3) and similarly defined counties with a large share of Latinos but fewer immigrants (type 5 and type 7) had a higher likelihood of preterm birth. A plausible explanation for this finding is that confining historically marginalized communities to under-resourced areas concentrates economic and social disadvantages, which impacts the level of social capital within counties [31].

Our results for disadvantaged suburban counties with a large Latino and immigrant presence may further illustrate this social and economic interaction. Latina mothers in these counties had a higher level of exposure to other Latinos which resulted in a significantly lower likelihood of preterm birth in fully adjusted models. However, increased dissimilarity between Latinos and white residents resulted in non-significant higher odds of this birth outcome. Thus, while increased exposure to other Latinos may reduce the likelihood of preterm birth, it may limit opportunities to establish protective bridging ties outside the coethnic community [31]. While our results deviate from research that suggests social support mitigates the harmful effects of living in materially deprived settings [9, 15], they align with others that find residing in economically constrained areas lessens the benefits of living among coethnic community members [9, 34, 35].

These effects may also be compounded by urban or suburban geography and the barriers residents face to establishing strong social and organizational ties in different areas [22, 26]. Although Latina mothers in all urban counties had higher odds of preterm births than their counterparts in affluent suburban areas, this likelihood was lower than that for residents in high economic disadvantage suburban counties with a large share of Latinos and immigrants. However, urban counties had, on average, higher levels of income inequality and fewer social associations than their suburban counterparts, which may explain the higher likelihood of preterm birth in these areas. It is also possible that despite the concentration of support services and resources in urban settings, Latina mothers may face economic or other barriers that limit their access.

We also found that other county and state-level characteristics increased the likelihood of preterm births. Specifically, Latina mothers in places that had more county-level immigration enforcement policies experienced a higher likelihood of preterm births. However, previous studies have not been unequivocal in linking county and state-level immigration enforcement or criminalizing immigrant policies to preterm births among Latinas [51–53]. In this study, we used a broader set of county-level immigration enforcement policies and focused only on suburban and urban counties where Latinas made up a larger share of the population. Future studies that apply this approach to other geographic scales and among other populations are needed.

A related finding was that Latina mothers who lived in states with more undocumented people experienced an increased likelihood of preterm births. Although it is difficult to decipher a likely undocumented population using NCHS birth records, extant research suggests that local jurisdictions—especially suburban areas—pass exclusionary immigrant policies as their undocumented population increases [54]. Such areas may also share other social and economic characteristics that influence birth outcomes [21, 22]. Future research may examine how contextual factors interact in diverse immigration policy settings to shape birth outcomes.

Taken together, our results suggest that residential contexts shape preterm birth among Latina mothers in varied ways. In considering the broader settings in which enclaves exist, we show that county-level social, economic, and geography-related factors interact to shape Latina mothers’ access to social support, economic opportunities, and health and public services. Since residential contexts may influence preterm birth risk in different ways, multi-layered strategies are needed. Amendable points of intervention might include establishing or strengthening incentive programs that increase the number of obstetric and primary care providers in places most in need of them [55]. Such efforts may help to ensure women and other birthing people have access to pregnancy-related services. In addition, policies that bolster economic conditions in local communities are also needed to contend with growing wage gaps and the resulting health effects of income inequality [56]. Studies that examine a broader set of health outcomes, including those that have potentially different mechanisms than the ones we discuss, are needed to substantiate these findings.

### Strengths and Limitations

This study had several strengths. We used a county-level measure that integrates social, economic, and geography-related characteristics and applied it to preterm birth—an area where research on enclave-health effects has often focused. We also considered additional community and structural-level factors, which allowed us to adjust for a diverse set of influences that likely operate at different scales. This study had nearly complete cases of birth records for Latina mothers living in the classified counties, which allowed us to investigate patterns in each county type and their associations with preterm birth. An added strength is that we build upon research on health outcomes in multiethnic suburban communities which, except for a few studies [57–59], have received limited attention in public health research.

However, our results should be reviewed with attention to specific limitations. This study was cross-sectional, which limits causal interpretations. Our exposure variable was a county-level measure that may not capture how social support, social networks, and other phenomena operate at the neighborhood level [22]. Nevertheless, the county may represent an appropriate geographic level for which to conceptualize and measure health-related effects, including those that stem from macro-level processes [22]. We were also unable to include rural counties because the original classification excludes such areas; so, the extent that these results vary across rural communities is unknown and a point for future investigations. Another limitation stems from our use of birth records, which limited our ability to control for other factors, like length of residence in particular areas.

## Conclusion

This study extends research on residential contexts and preterm births among Latina mothers in the US and provides evidence that county-level social, economic, and geography-related characteristics may combine to disparately influence PTB risk. Our findings may help to refine study questions, interventions, and policies toward creating healthy environments for expectant mothers. Understanding the influence of residential contexts on preterm births among Latina mothers requires considering the broader social, economic, and geography-related settings in which enclaves exist. A focus on context may help to better align research on enclave-health effects with the health and welfare goals of county governments.

## Data Availability

The natality files used in this study are restricted-use and not publicly available; all other datasets are publicly accessible. See a detailed list of data sources and corresponding variables in Appendix Table 5.

## Appendix

Table 5

**Table 5:** Study variables, data sources, and publishers

Variable	Data source	Publisher
1. Preterm birth	U.S. Natality data (2017–2018)	National Center for Health Statistics (NCHS), Centers for Disease Control and Prevention (CDC)
2. Mother’s nativity		
3. Mother’s Latino origin		
4. Parity		
5. Smoking		
6. Risk factors		
7. Age		
8. Education		
9. Marital status		
10. Prenatal care initiation		
11. Health insurance		
12. County classification	American Community Survey (ACS) (2014–2018) and the NCHS Urban–Rural Classification Scheme for Counties	U.S. Census Bureau and the NCHS, CDC
13. Isolation index^a^	ACS (2014–2018)	U.S. Census Bureau
14. Dissimilarity index^a^		
15. Population density (county)		
16. % population change since 2000 (state)	Calculated with the decennial survey (2000) and ACS (2014–2018)	
17. Income inequality (county)	County Health Rankings (2019–2021)	University of Wisconsin Population Health Institute
18. Social associations (per county population)		
19. The ratio of the county population to primary care physicians		
20. The ratio of the county population to other primary care providers		
21. % rural population (county)		
22. Immigration enforcement policies (county)	National Map of Local Entanglement with ICE (2019)	Immigrant Legal Resource Center
23. % of county electorate voting Republican	County Presidential Election Returns (2016)	MIT Election Lab
24. County public health expenditure	Census of Governments	U.S. Census Bureau
25. State covers lawful immigrants without 5-year wait	Medicaid and CHIP Eligibility, Enrollment, and Cost Sharing Policies as of January 2019	Kaiser Family Foundation
26. % undocumented foreign-born population in state	Unauthorized Immigrant Population Profiles (2014–2018 ACS)	Migration Policy Institute

Note: ^a^Expressed as a weighted average at the county level

Table 6

**Table 6:** Pairwise comparisons across county-type classification dimensions

Comparison		Latino density	Immigrant concentration	Economic disadvantage	Geography	Odds ratio	*p*-value
Variable	County type						
Latino ethnic density	10 vs. 2	High	High	Low	Suburban	0.92	0.00
		Med	High	Low	Suburban		
	9 vs. 1	High	High	High	Suburban	0.79	0.00
		Med	High	High	Suburban		
Immigrant concentration	6 vs. 2	High	High	Low	Suburban	0.97	0.29
		High	Low	Low	Suburban		
	5 vs. 1	High	High	High	Suburban	0.89	0.00
		High	Low	High	Suburban		
	7 vs. 3	High	High	High	Urban	1.05	0.18
		High	Low	High	Urban		
Economic disadvantage	2 vs. 1	High	High	Low	Suburban	0.87	0.00
		High	High	High	Suburban		
	4 vs. 3	High	High	Low	Urban	0.95	0.00
		High	High	High	Urban		
	10 vs. 9	Med	High	Low	Suburban	1.01	0.70
		Med	High	High	Suburban		
	6 vs. 5	High	Low	Low	Suburban	0.94	0.02
		High	Low	High	Suburban		
Geography	4 vs. 2	High	High	Low	Suburban	0.96	0.06
		High	High	Low	Urban		
	3 vs. 1	High	High	High	Suburban	0.88	0.00
		High	High	High	Urban		
	7 vs. 5	High	Low	High	Suburban	1.04	0.38
		High	Low	High	Urban		

Note: The odds ratio represents the differences between the log odds comparing preterm births across county-type pairs. The log odds were exponentiated to derive the odds ratio
